# Nanoemulsion-based dissolving microneedle arrays for enhanced intradermal and transdermal delivery

**DOI:** 10.1007/s13346-021-01107-0

**Published:** 2021-12-22

**Authors:** Muhammad Iqbal Nasiri, Lalitkumar K. Vora, Juhaina Abu Ershaid, Ke Peng, Ismaiel A. Tekko, Ryan F. Donnelly

**Affiliations:** 1 School of Pharmacy, Medical Biology Centre, Queen’s University Belfast, 97 Lisburn Road, Belfast, BT9 7BL UK; 2 Department of Pharmaceutics, Hamdard Institute of Pharmaceutical Sciences, Hamdard University, Islamabad, Pakistan

**Keywords:** Nanoemulsion, Dissolving-microneedles, Synergistic effect, Amphotericin B, Transdermal delivery

## Abstract

The development of dissolving microneedles (DMN) is one of the advanced technologies in transdermal drug delivery systems, which precisely deliver the drugs through a rapid dissolution of polymers after insertion into the skin. In this study, we fabricated nanoemulsion-loaded dissolving microneedle (DMN) arrays for intradermal and transdermal drug delivery. For this task, model drug (amphotericin B, AmB)-loaded nanoemulsion (NE) were prepared by the probe-sonication method. AmB-loaded-NE was prepared using Capmul MCM C-8 EP/NF, Tween^®^ 80, poly(vinyl alcohol) (PVA-10 kDa), and poly (vinyl pyrrolidone) (PVP-360 kDa or K29/32) by using SpeedMixer™, followed by probe-sonication and evaluated for particle size and polydispersity index (PDI). Transmission electron microscopy (TEM) was also used to assess the particle size before and after DMN casting. AmB-NE embedded DMN arrays were found to be strong enough, revealed efficient skin insertion, and penetrated down to the fourth layer (depth ≈ 508 μm) of Parafilm M^®^ (validated skin model). Ex vivo skin deposition experiments in full-thickness neonatal porcine demonstrated that after 24 h, AmB-NE-DMN arrays were able to deposit 111.05 ± 48.4 µg/patch AmB into the skin. At the same time, transdermal porcine skin permeation studies showed significantly higher permeability of AmB (29.60 ± 8.23 μg/patch) from AmB-NE-DMN compared to MN-free AmB-NE patches (5.0 ± 6.15 μg/patch) over 24 h. Antifungal studies of optimized AmB-NE-DMN, AmB-loaded discs and drug-free DMN against *Candida albicans*, confirmed the synergistic activity of Campul-MCM C-8, used in the nanoemulsion formulation. This study establishes that nanoemulsion based dissolving microneedle may serve as an efficient system for intradermal as well as transdermal drug delivery.

**Graphical abstract:**

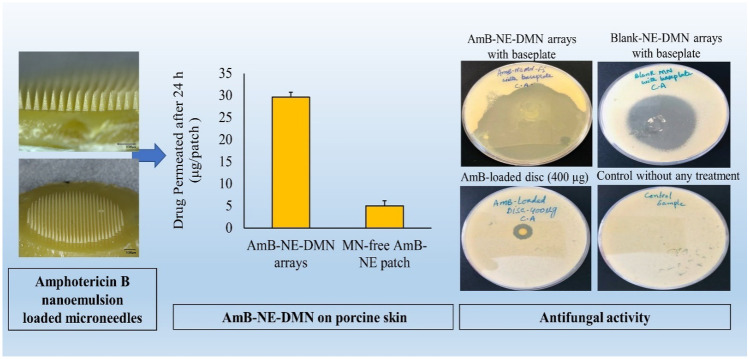

## Introduction

Transdermal drug delivery is beneficial for the administration of therapeutic molecules, as it bypasses the first-pass metabolism associated with oral administration [1], depending upon the size of molecules [2]. Microneedles (MNs) are micron-scale devices/projection arrays (50–900 μm), which can painlessly penetrate the outermost layer of the skin (*stratum corneum)* to facilitate intradermal delivery of drugs and vaccines [3, 4]. MN arrays have significantly filled the gap between the transdermal patches and pain-causing injections in the last two decades. MNs can produce a transport pathway for drug molecules by overcoming the skin’s barrier properties and have been revealed to be suitable for self-administration by patients [5]. Among different types of MN, dissolving microneedles (DMN) are developed by employing biodegradable, water-soluble polymers that entirely degrade or dissolve in the skin and the whole drug released beneath the *stratum corneum*. MN also reduces any likely risk of biohazardous sharps waste, and potential sustained delivery of small molecules is possible [6, 7].

It is quite challenging to develop a DMN for lipophilic drugs because of immiscibility issues of hydrophobic drug particles into an aqueous polymeric phase of DMN formulation. Therefore, DMN formulation from lipophilic drugs causes a drug loading and content uniformity problem, which may be overcome by using organic solvents to improve the solubilization. However, the use of organic solvents can also reduce the strength of DMN, as they can produce pores or residual solvents in the hydrophilic polymer matrix [8–11]. Lipophilic drugs are more potential candidates for developing topical formulations because of their significant benefits, such as high permeability across the cellular lipid membrane, enhanced absorption, and metabolism, and greater therapeutic achievement over hydrophilic drugs [12–14]. Another fascinating system explained formerly in the literature was nanoemulsion (NE). NE is a type of dispersion of water-in-oil or oil-in-water, stabilized by a surfactant, with a small droplet size (< 500 nm). NEs are heterogeneous emulsified, kinetically stable drug carrier systems, generally formed by high-energy methods [15, 16]. Various oils have been used in NE, like, isopropyl myristate, castor oil, triacetin, soybean oil, sesame oil, coconut oil, Capmul PG8, triacylglycerides, vitamin E, and clove oil [17, 18].

NE has many advantages, like easy fabrication, high stability, increased drug solubility, and enhanced bioavailability, particularly for hydrophobic drugs [18, 19]. Despite the certain benefits, NEs have some limitations of low viscosity and spreadability, thus restricting its topical use. Few studies suggested adding gelling agents such as chitosan, Carbopol^®^ family, methylcellulose, and poloxamer 407 to change formulations' physical state and pharmacokinetic properties [20, 21].

Formerly, various studies have been reported related to the fabrication of AmB-loaded nanoemulsion to treat topical fungal infections [17, 22–24]. Presently, fungal infections are one of the most significant public health issues. Particularly among immunocompromised patients (AIDS) and patients receiving immunosuppressive chemotherapy or transplantation, infections caused by *Candida* spp. have increased in the last 30 years [17]. Mucormycosis, also known as black fungus, is a serious but rare fungal infection caused by a group of molds (mucormycetes) that live throughout the environment, particularly in soil, plants, leaves, fruits, vegetables, air, rotten woods, and even in the mucus of healthy people. It affects the sinus, brain, and lungs and can be life-threatening, particularly in diabetic or severely immunocompromised individuals [25, 26]. Mucormycosis is another secondary complication added in COVID-19 that has arisen as a deadly complication. In March 2021, approximately 41 cases of COVID-19-associated mucormycosis were reported worldwide, and 70% were from India [26]. This black fungus was continued to spread in India during the deadly second wave of the COVID-19 infection, and approximately 28,200 cases mucormycosis have recorded across the country as of 7 June 2021. Roughly 86% of patients with deadly fungal infections had contracted with a history of COVID-19. In this critical situation, a doctor recommends amphotericin B (AmB) to overcome the black fungus. AmB is a broad-spectrum antifungal and antiprotozoal macrolide polyene antibiotic, very effective against a broad range of pathogenic and opportunistic fungal species. It is used in the treatment of the most frequent systemic and cutaneous fungal infections caused by *Candida* spp. and *Aspergillus* spp. [27]. It also shows activity against cutaneous leishmaniasis (parasitic disease) and mucocutaneous leishmaniasis [28].

The AmB can not be absorbed directly through the skin [29]; therefore, several kinds of micro-and nano-sized drug delivery systems were described in the literature previously, such as liposomes [30] and solid lipid nanoparticles [31]. Therefore, we present a simple and innovative in-situ AmB NE generation in DMN polymeric hydrogel to prepare the AmB NE-loaded DMN arrays that could synergetically improve the intradermal delivery of AmB. Initially, AmB based NE was prepared by the probe-sonication method in polymeric hydrogel and then characterized for droplet size, PDI, and zeta potential and, subsequently, cast into DMN by single-step centrifugation method. This delivery system was designed, specifically focusing on determining the possibility of using a novel DMN system to facilitate intradermally and transdermal delivery of AmB loaded NE. The developed AmB-NE-DMN system was then evaluated for ex vivo intradermal neonatal porcine skin permeation and drug deposition studies.

## Materials and methods

### Materials

Amphotericin B was procured from Enke Pharma^®^ (Cangzhou Enke Pharma-tech co, Ltd, Hebei, China). Olive oil and poly(vinyl alcohol) (PVA) of molecular weight 9000–10,000 Da were purchased from Sigma-Aldrich (Chemie GmbH, Japan). Soyabean oil and oleic acid were purchased from Sigma-Aldrich (Chemie GmbH, USA). Sesame oil was purchased from Sigma-Aldrich (Chemie GmbH, Mexico). Castor oil was purchased from Ransom Natural Ltd (Hitchin, England). Campul PG-8/NF^®^ (propylene glycol monocaprylate) and Campul MCM C-8 EP/NF (glyceryl monocaprylate) were kindly gifted by ABITEC Corporation (Wisconsin, USA). Polyoxyethylene sorbitan monooleate (Tween^®^80) was obtained from VWR Chemical^®^ (Solon, Ohia, USA). Soybean Lecithin was supplied from Tokyo Chemical Industry co, Ltd (Tokyo, Japan). Poly(vinylpyrrolidone) (PVP) of molecular weight 580,000 Da (K-29/32) was purchased from Ashland Industries (Wilmington, USA). Purified water utilized in all experiments was obtained from ELGA^®^ DV 25, Purelab Option, water purification System (ELGA-Q, USA). All the other chemicals and reagents were of analytical grade.

### Methods

#### Solubility assessment of AmB

The solubility of AmB in different oils, lipids, fatty acids, surfactants, and water was assessed by dissolving an excess amount of AmB in 1 g of each component. The Eppendorf tubes were vortexed for 1 min and kept in an Eppendorf tube shaker at 37 ± 2 °C for 48 h. After that time, the Eppendorf tubes were centrifuged (14,800 rpm, 20 min), and then 0.3 g supernatant of each sample was filtered and diluted in 1 mL mixture of dimethyl sulfoxide (DMSO):methanol (50:50, v/v). Afterward, each sample was again filtered through 0.2 µm syringe filter (Agilent Technologies, USA). The solubility of AmB was quantified by using a validated high-performance liquid chromatography (HPLC) technique. NE was prepared by titration of homogamous mixtures of an oil phase and water phase. For this task, different combinations of oil_mix_ (Campul-MCM C-8 & DMSO) and surfactant at different percentage ratios (90:10, 80:20, and 70:30) were mixed with water phase to delineate the boundaries of emulsification. The aqueous phase consisted of 40% (w/w) PVA (MW 9000–10,000 Da) and 40–60% (w/w) PVP (MW 58,000 Da, K-29/32) as co-surfactant as well as DMN forming agent.

#### Preparation of AmB nanoemulsion

**Fig. 1:**
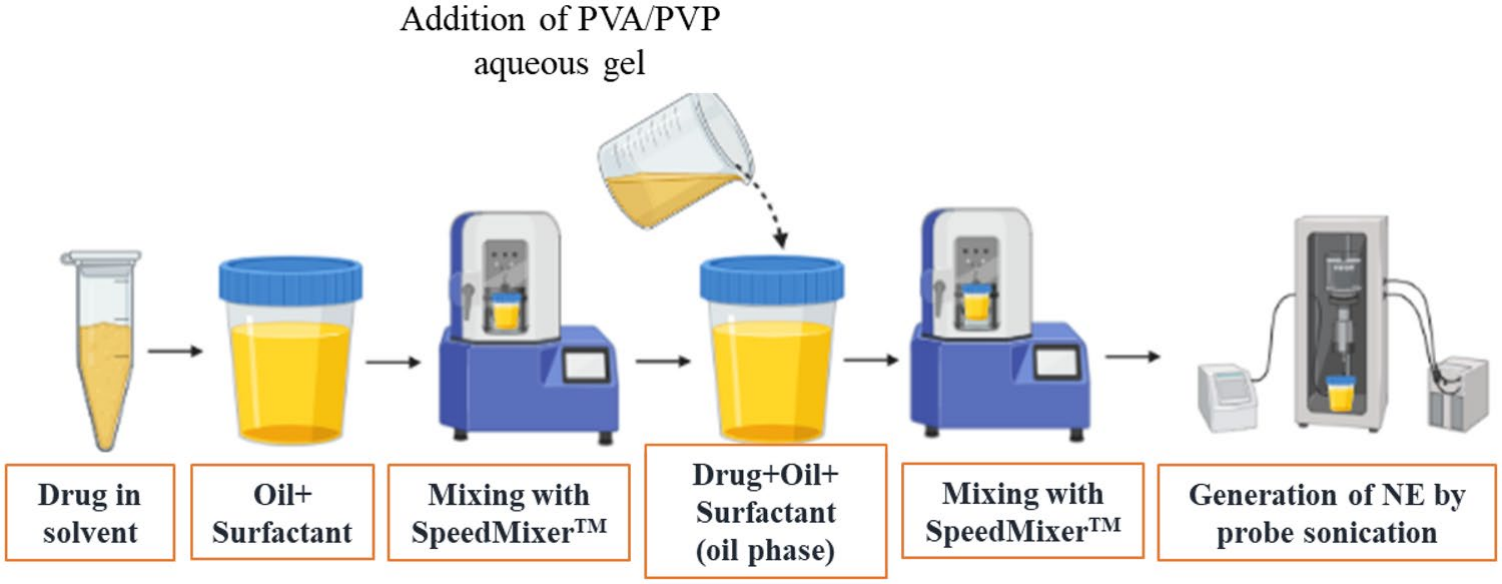
Schematic representation of AmB-loaded NE preparation

Figure 1 shows the schematic representation of fabrication of NE by probe sonication method with a modification as reported previously [16].

The AmB-NE was developed by mixing the oil phase with the aqueous phase using SpeedMixer™ (DAC 50, FVZ-K, Hauschild Engineering, Germany), followed by probe sonication (QSonica, Q125, LLC, Church Hill, Newtown, USA) for 6 min at 100% amplitude and 125 W, with 10 sc pulses on and 5 s pulse off [32]. The AmB-NEs were prepared by placing the container under an ice bath to prevent the rising temperature during sonication. In the present study, Capmul MCM C-8 EP/NF was selected as an oil phase due to maximum drug solubility and inherent antifungal activity, whereas Tween^®^ 80 was chosen as a surfactant due to having maximum drug solubility and good emulsification capacity. PVA and PVP were used as co-surfactant as well as DMN-forming polymers. A fixed amount of AmB (40 mg) was dissolved entirely in 0.5 g of DMSO and was mixed with oil (Capmul MCM C-8) and surfactant using SpeedMixer™. Next, the AmB containing oil phase was completely dispersed in an aqueous phase containing PVA (40% w/w) and PVP (40 – 60% w/w) by using probe sonication for 6 min at 100% intensity, under an ice bath. The composition of formulations is presented in Table 1.

**Table 1:** Formulation composition of AmB-loaded NE

Formulation code	Oil phase (%, w/w)	Aqueous phase (%, w/w)	Aqueous Phase (%, w/w)				Nominal conc. of AmB (mg/mL)
			40% PVA, K-10 (%, w/w)	40% PVP, K-29/32 (%, w/w)	50% PVP, K-29/32 (%, w/w)	60% PVP, K-29/32 (%, w/w)	
AmB-NE-F1	10	90	40	50	0	0	4
AmB-NE-F2	10	90	20	70	0	0	4
AmB-NE-F3	12.5	87.5	12.5	75	0	0	5
AmB-NE-F4	12.5	87.5	12.5	0	75	0	5
AmB-NE-F5	12.5	87.5	12.5	0	0	75	5

*Oil phase containing Campul MCM-C8: DMSO:Surfactant (4:5:1) and aqueous phase containing 12.5, 20, or 40% of PVA (K-10) 40% solution; 50, 70, or 75% PVP (K-29/32) 40% solution; 75% PVP (K-29/32) 50% solution; and 75% of PVP (K-29/32) 60% solution

#### Characterization of AmB-NE

The fabricated AmB-NE formulations were evaluated for different physical parameters such as droplet size and polydispersity index (PDI) by dynamic light scattering (DLS) technique using NanoBrook Omni (Brookhaven Instrument, Holtsville, USA) at a temperature of 25 °C with a scattering angle of 90° [33, 34]. Particle size by dynamic light scattering gives the hydrodynamic radius of particles and the PDI, which is a measure of the width of the size distribution. Samples were diluted in water to a suitable concentration (1.0%, v/v) prior to analysis. Finally, the zeta potential (mV) of the AmB-NE formulations was determined using the same instrument by applying an electric field across the NE solutions using the phase analysis light scattering (PALS) technique to establish the electrophoretic mobility of charged, nano-dispersion. All experimental runs were performed in triplicate to obtain mean data.

#### Morphological studies using TEM

The morphology (droplet size and shape) of drug-loaded NE-F5, blank-NE and AmB-NE-DMN-F5 formulations were assessed using transmission electron microscopy (TEM) technique (JEM-1400Plus; JEOL, Tokyo, Japan). Each formulation (1-mL) was diluted with water (100-folds) followed by continuous stirring to ensure homogeneous mixing. Then, a drop of the diluted sample was put on a copper grid previously coated with carbon film, and the excess sample was removed from the grid using a non-shedding filter paper. Finally, for ease of scanning during analysis, negative staining agent (uranyl acetate solution) was dropped on the copper grid [34]. The excess agent was removed with filter paper. Before TEM scanning and image analysis, the grid was dried in the open air at ambient temperature (25 °C).

#### Stability studies

The AmB-NE formulation that showed better characteristics (AmB-NE-F5) was chosen for stability study for 15 days. Fresh AmB-NE formulation was prepared using the same method as discussed earlier. The selected formulation was stored in a sealed plastic container and placed under two different conditions: 4 ± 2 °C (under refrigeration) and 25 ± 2 °C (room temperature). Various stability-indicating parameters such as appearance (by visual inspection), droplet size, polydispersity index, and zeta potential were evaluated on 0, 7, and 15 days.

#### Fabrication of AmB NE-loaded DMN

**Fig. 2:**
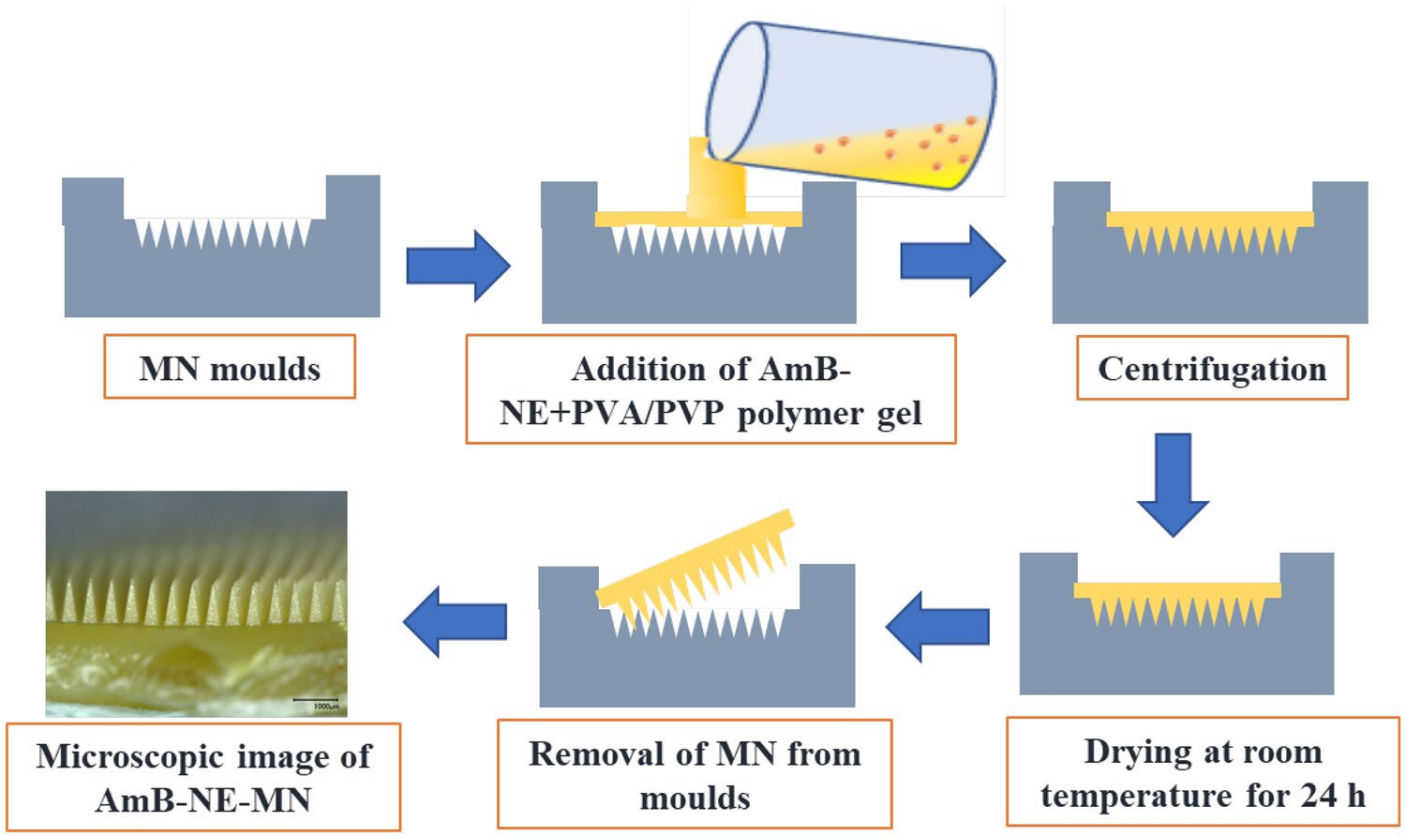
Schematic representation of fabrication of AmB-NE-DMN arrays

The fabrication of AmB nanoemulsion-loaded dissolving microneedles (AmB-NE-DMN) is summarized in Fig. 2. AmB-NE-DMN were prepared in a single-step centrifugation process of microneedle casting (DMN and baseplate were prepared using the same AmB-NE). The silicone MN molds were designed with 600 arrays per 0.75 cm^2^ area. These molds with microneedle heights of 700 μm, base width of 300 μm, and interspacing of 15 μm. Briefly, AmB-NE-DMN were fabricated by pouring AmB-NE onto the top surface of the MN moulds, and the moulds were centrifuged at 3500 rpm for 20 min and then allowed to dry for 24 h at room temperature and then kept in an oven at 37 ± 2 °C for further drying of 24 h. MN arrays were then removed from the moulds and evaluated for needle formation and mechanical strength [32, 35].

#### Post-microneedle formation evaluations

The optimized AmB-NE-MN-F5 was dispersed completely in water, and after appropriate dilution, droplet size, PDI, and zeta potential were evaluated using NanoBrook Omni (Brookhaven Instrument, Holtsville, USA), at 25 °C. The amount of drug-loaded into DMN was assessed by high-performance liquid chromatography analysis.

#### HPLC analysis

Using the validated analytical method, the quantitative analysis of AmB in AmB-NE-MN was performed by reversed-phase HPLC method (Agilent 1200® Binary Pump, Agilent 1200^®^, Standard Autosampler, Agilent 1200^®^ Variable Wavelength Detector; Agilent Technologies UK Ltd., Stockport, UK) with Phenomenex, ODS-C18 (150 mm × 4.6 mm, 5 µm particle size) column. The mobile phase consisted of buffer and organic phases (35:65, v/v). Buffer phase composed of ethylenediaminetetraacetic acid disodium salt dihydrate (2.5 mM), and organic phase consisted of a mixture of acetonitrile, methanol, and tetrahydrofuran (THF) (41:18:10, v/v). The HPLC system was run with isocratical method at an ambient temperature at a flow rate of 1 mL/min with UV detection at 385 nm. The stock standard solution was prepared by dissolving 10 mg of AmB in 2 ml of DMSO and then diluted with methanol to get 100 µg/mL. The linearity was determined from seven working standard solutions of 0.25, 0.5, 1, 2, 4, 8, and 16 µg/mL in methanol. The correlation (*r*^2^), intercept, and slope of the standard curve was calculated. The peak areas of samples were calculated, and the concentrations of AmB in the formulation samples were determined from the standard curve. This linearity was performed in triplicate [13, 36].

#### Drug content analysis of AmB-NE-DMN

The drug content in AmB-NE-DMN was quantified by dispersing DMN patch into a glass vial containing 10 mL water, sonicated for 15 min, and diluted with 10 mL methanol, followed by sonicated for a further 15 min. Then, 200 μL was taken into 1.5 mL tubes and mixed with 0.8 mL acetonitrile to precipitate PVP polymer while the drug remained dissolved [37]. This dispersion was centrifuged at 14,800 rpm for 10 min, and the supernatant was collected for HPLC analysis. These experiments were performed in triplicates.

#### Microscopic analysis AmB-NE-DMN

The surface morphology and shape of AmB-NE-DMN were examined by using optical and scanning electron microscopy. A Keyence VHX-700F Digital Microscope (Keyence, Osaka, Japan) and TM3030 benchtop scanning electron microscope (SEM) (Hitachi, Krefeld, Germany) were used for evaluation. The SEM was used in low vacuum mode at a voltage of 15 kV [38].

#### Mechanical strength and Parafilm M^®^ insertion properties of AmB-NE-DMN

To assess the strength and insertion properties of the AmB-NE-DMN, a TA.XT-Plus Texture Analyzer (Stable Microsystem, Haslemere, UK) was used in compression mode. The heights of DMN were initially determined using a Leica EZ4 W digital microscope (Leica Microsystems, Wetzlar, Germany). Afterward, AmB-NE-DMN were adhered to the removable cylindrical probe using double-sided adhesive tape and processed against a flat metal block at a rate of 0.5 mm/sec for 30 s and the force applied was 32 N [39]. The heights of DMN were again measured using the Leica EZ4 W-microscope (Leica Microsystems, Wetzlar, Germany). The reduction in the heights of DMN after application of compression force was measured in percentage.

The insertion properties of the DMN were assessed using Parafilm M^®^ (Bemis Company Inc., Soignies, Belgium), a flexible thermoplastic sheet made of olefin-type material. Parafilm M^®^ was used as a skin simulant for DMN insertion studies in order to determine the mechanical strength of the AmB-NE-DMN as reported previously [40–42]. Prior to the test, the initial heights of DMN were microscopically measured. Eight layers of Parafilm M^®^ (1 mm thickness) were folded and placed over the surface of the steel block, followed by the attachment of AmB-NE-DMN to the movable texture analyzer’s probe. Then, AmB-NE-DMN was inserted at a speed of 1.19 mm/s, with a force of 32 N for 30 s. Afterward, the DMN was detached from the Parafilm M^®^ layer, and then each Parafilm M^®^ layer was examined microscopically to count the number of holes per layer. The heights of DMN were again evaluated using a Leica EZ4-W digital microscope to check any reduction in the length of needles.

#### Excised neonatal porcine skin insertion studies by optical coherence tomography

The insertion of the AmB-NE-DMN was evaluated in-situ using full-thickness neonatal porcine skin, which is a simulated model of the human skin [43]. The skin was obtained from stillborn piglets and excised within 24 h of birth using an electric dermatome (Integra Life Sciences™, NJ, USA). The skin was then wrapped in aluminum foil and stored at -20 °C until use. After thawing in phosphate-buffered saline (PBS) (pH 7.4), the skin was carefully shaved using a razor and washed with PBS before use. The skin surface was dried using tissue paper and placed dermis side down on a dental wax sheet to give support, and the underside of the skin was bathed in PBS (pH 7.4) at 37 °C for 30 min to equilibrate. Optical coherence tomography (OCT) images were captured immediately upon insertion using an OCT Microscope (EX1301, Michelson Diagnostics Ltd., Kent, UK), to assess the successful insertion of the microarrays patch into the Parafilm M^®^ layers as well as neonatal porcine excised skin [44, 45]. DMN patches were applied manually for 30 s. OCT was used with a laser center wavelength of 1305 ± 15 nm to facilitate real-time high-resolution imaging of upper skin layers. The OCT images were examined using the imaging software ImageJ^®^ (National Institute of Health, Bethesda, USA). The scale of image files obtained was 1.0 pixel = 4.2 μm, thus allowing accurate measurements of the depth of MN penetration, the distance between the MN baseplate and the *stratum corneum* [46].

### Dissolution studies of AmB-NE-DMN into excised porcine skin

AmB-NE-DMN were also inserted manually for 30 s into the center of the skin, and a stainless-steel weight (12 gm) was placed on top to ensure the arrays remained in place. Afterward, DMN arrays were taken out from the skin at various time points (5, 15, 25, and 40 min) and immediately viewed under a Leica EZ4 W digital microscope to observe the dissolution of DMN into the skin. Separately, AmB-NE-DMN was again inserted manually for 30 s into the skin, and immediately OCT images were taken to observe the penetration of DMN arrays into excised porcine skin.

### Ex vivo deposition studies of AmB-NE-DMN into excised porcine skin

In this study, full-thickness neonatal porcine skin was used as a human skin model to study the insertion of AmB-NE-DMN. The skin (approximately 1.2 mm thickness) was obtained from stillborn piglets and excised within 24 h of birth using a scalpel [45]. The skin was then wrapped in aluminum foil and stored at − 20 °C until use. After thawing in phosphate-buffered saline (PBS) (pH 7.4), the skin was carefully shaved using a razor and washed with PBS before use. The skin surface was dried using tissue paper and placed dermis side down on a dental wax sheet to give support, and the underside of the skin was bathed in PBS (pH 7.4) at 37 °C for 30 min to equilibrate. After insertion of MN patch, a cylindrical 12.0 g stainless steel weight was placed onto the top of the MN arrays patch to prevent MN expulsion and placed inside the oven at 37 °C ± 2 °C for 24 h. The tissue sample, taken from that portion of the skin where the AmB-NE-MN had been inserted, were obtained using a scalpel. The samples were cut into small pieces and transferred into 2-mL Eppendorf tubes. For simultaneous disruption and homogenization of tissues and to extract the amount of AmB that permeated into the skin, 1 mL DMSO was added, and bead milled using TissueLyser LT (QIAGEN^®^, UK) for 15 min. Collected the sample in a glass vial and again added 1 mL DMSO and ran for 15 min more. Transferred the whole sample in a glass vial and diluted with 2 mL acetonitrile and sonicated for 15 min. Next, 1 mL samples were taken in 1.5 mL tubes and then centrifuged at 14,800 rpm for 10 min. Supernatants were analyzed using the validated HPLC method.

### Ex vivo porcine skin permeation of drug from AmB-NE-DMN

The ex vivo dermatomed neonatal porcine skin was investigated using Franz diffusion cells as reported previously [47] and trimmed to a thickness of 350 μm using an electric dermatome (Integra Life Sciences™, NJ, USA) to study the permeation of AmB across the skin. The skin was stored in aluminium foil at − 20 °C until further use. Before use, the skin was bathed in PBS to thaw and carefully shaved. Sections of skin were cut by scalpel equal to the diameter of the Franz cell donor compartments and carefully affixed to the donor compartment on the *stratum corneum* side using cyanoacrylate super glue (DIY, Willenhall, UK), rendering the stratum corneum available for DMN application. AmB-NE-MN were then inserted using manual pressure for 30 s applied to the MN baseplate. For comparison, MN-free AmB-NE patches were also applied over the skin. A cylindrical 12.0-g stainless steel weight was placed onto the top of the DMN arrays patch to prevent DMN expulsion, and the donor compartment of the apparatus was clamped onto the receiver compartment and sealed using Parafilm M^®^. The receiver compartment contained 12 mL PBS with 1% (w/v) sodium lauryl sulphate to maintain the solubility of the drug in the receiver compartment. Syringes (1.0 mL) with long needles were used to remove 200 μL from the Franz cell contents at a different time interval (up to 24 h) and the same volume of prewarmed PBS was replaced to the receptor medium. Samples were stored in 0.5-mL polystyrene tubes and were centrifuged for 15 min at 14,800 rpm using an Eppendorf Minispin centrifuge (Eppendorf UK Ltd, Stevenage, UK). All the samples were analyzed for drug content using HPLC. Permeation from control AmB-NE was performed in the same manner, except instead of inserting a DMN array, a needle-free patch of the same dimensions and formulation was placed on top of the skin, followed by the stainless-steel weight.

### In vitro antifungal activities

Disk diffusion method (known as Kirby-Bauer) was adopted with modification as per previously published work [48], to determine the antifungal activity of AmB against *Candida albicans* (*C. albicans* NCYC 610, stock of Microbiology Laboratory, School of Pharmacy, Queen’s University Belfast, UK). Concisely, fungi-culture was prepared freshly, and the viable colony-forming units (CFUs) concentration/cell density was demonstrated approx. 6 × 10^6^ CFU/mL. Sabouraud dextrose agar (SDA) media was prepared and sterilized as per the manufacturer’s instructions. Now, 5 mL soft SDA was heated at 50 °C and mixed with 1 mL fungi solution. The mixture was vortexed and poured on the surface of a plate, having solidified agar-media, while rotating the plates to ensure even distribution. After inoculation, the surface of the agar was allowed to cool and dry for 15 min. Briefly, seven groups were set and named as A, B, C, D, E, F, and G. For example, A = tips of AmB-NE-DMN-F5 arrays (approx. 35 µg of AmB), B = AmB-NE-MN-F5 arrays with a baseplate (427 µg), C = tips of blank-NE-MN arrays, D = complete blank-NE-DMN arrays with baseplate, E = AmB-loaded disc (400 µg), F = AmB-loaded disc (10 µg), and G = control inoculated plate, without any treatment. The whole process was performed under aseptic conditions (*n* = 4). Afterward, using sterile forceps, AmB-NE-MN-F5, blank-NE-DMN (AmB-free) and AmB-loaded discs were placed on the agar surface and slightly pressed down to insert the MN into the medium. After applications of MN and disks, plates were incubated at 37 °C in an incubator for 72 h. After this incubation period, the plates were examined, and the zone of inhibition was measured (mm).

## Results and discussion

Due to the *stratum corneum* barrier, effective transdermal drug delivery to treat subcutaneous mycoses and fungal infection remain challenging [49]. To resolve these issues, DMN patches were designed with drug-lipidic nanosystem-loaded arrays in order to deliver the drug transdermally as well as intradermally. DMN serves as drug reservoirs and is self-implanted subcutaneously to release antifungal drugs locally and sustainably without producing systemic side effects. DMN are affixed on the skin surface that painlessly pierces the epidermis, creating microscopic aqueous pores through which drugs diffuse to the dermal microcirculation [13, 36].

### Solubility assessments

The solubility assessment of AmB in the specified oils, lipids, fatty acid, surfactants, and water are provided in Table 2.

**Table 2:** Solubility of AmB in different oils, lipids, fatty acid, surfactants, and water

S. no	Oil/surfactants	Solubility (µg/mL) Mean, SD (*n* = 3)
1	Castor oil	55.63 ± 0.05
2	Sesame oil	46.32 ± 0.04
3	Olive oil	23.86 ± 0.06
4	Soyabean Oil	113.64 ± 0.07
5	Campul PG8/NF	27.88 ± 0.04
6	Campul MCM C-8 EP/NF	180.18 ± 0.01
7	Oleic acid	30.63 ± 0.02
8	Lecithin	175.25 ± 0.07
9	Tween 80	197.01 ± 0.08
10	Water	39.70 ± 0.01

The solubility of AmB in the mentioned components was carried out to select the excipients with maximum solubilizing capacity. AmB has intensely low oral bioavailability (0.3%), poor water solubility, and less biological membrane permeability due to its high molecular weight (924 Da) and a long-chain hydrophobic portion in its molecular structure [32]. These characteristics of AmB are challenging for formulation scientists to deliver drugs adroitly and economically. Based on study outcomes, Campul-MCM C8 (glyceryl monocaprylate as oil phase) and Tween-80 (as surfactant) were chosen as components of NE due to their maximum drug solubility. Moreover, Campul-MCM C8 has antifungal activity as reported previously [23], thus can also produce a synergistic effect with AmB. It is classified as non-hazardous according to the OSHA hazard communication standard and GHS/EU CLP classification. Among these components, Campul-MCM C8 and Tween-80 revealed 180.18 ± 0.01 and 198.80 ± 0.08 µg/mL as maximum solubility values, respectively. Overall result depicted that the lipophilic nature of the drug helps to solubilize in the lipids.

NE were prepared using different combinations of oil_mix_ (Campul-MCM C8 and DMSO) and surfactant at different percentage ratios (90:10, 80:20, and 70:30) to delineate the boundaries of emulsification. It is an established fact that the minimum concentration of surfactant is required for the selection of optimized formula from the phase diagram [17].

### Preparation and optimization of AmB-NE

AmB-NE was prepared by using SpeedMixer™, followed by probe sonication as summarized in Fig. 1 [32]. The AmB-NE was developed by mixing the oil phase with the aqueous phase using SpeedMixer™, followed by probe sonication under an ice bath to prevent the rising temperature during sonication. Sonication was carried out at 100% of amplitude for 6 min with 10-s pulse-on and 5-s pulse-off mode. A pseudo-ternary phase diagrams were delineated using a different combination of oil_mix_ (Campul-MCM C8 and DMSO) and surfactant at different percentage ratios, mixed with water phase to obtain the boundaries of emulsification. Moreover, it was observed that a high concentration of surfactant in the NE decreased the strength of DMN arrays. Hence, in the current study, the least concertation of surfactant (90:10) was chosen to use in the NE formulations.

The formulation composition of AmB-NE has been presented in Table 2. Several formulations (AmB-NE-F1 to AmB-NE-F5) were prepared to optimize the concentration of PVA and PVP in order to achieve a good penetration of DMN into parafilm M^®^ as well as excised porcine skin. This optimization was also based on droplet size, PDI, zeta potential, mechanical strength, and maximum insertion capability of DMN. For further evaluation and characterization, the developed formulations were stored at room temperature.

### Characterization of AmB-NE

All formulations were characterized for droplet size, PDI, and zeta potential, as illustrated in Table 3. Sonication was carried out at 100% of amplitude for 6 min, which was found to be the optimal condition to attain a mean particle size of less than 350 nm and 0.28 PDI values for all formulations. The NE was optically homogenous, viscous, monophasic appearance, and yellowish without any precipitates. The optimized NE (AmB-NE-F5) showed a droplet size of 296.65 ± 4.74 nm, PDI of 0.192 ± 0.011, and zeta potential of − 24.90 ± 1.05. These results were indicative of the presence of nano-scale droplets and a monophasic system. NE is a thermodynamically stable formulation with nano-scale globular size, which is very important for effective activity against fungal strains. Thus, globular size and size distribution and zeta potential are treated as crucial parameters for the efficacy and stability of NE formulation.

**Table 3:** Characterization of AmB-NE (mean ± SD (*n* = 3)

Formulations	Droplet size (nm)	PDI	Zeta potential (mV)
AmB-NE-F1	211.12 ± 5.07	0.300 ± 0.009	− 25.16 ± 0.59
AmB-NE-F2	244.42 ± 3.59	0.251 ± 0.015	− 28.14 ± 0.94
AmB-NE-F3	314.06 ± 6.36	0.258 ± 0.025	− 25.86 ± 0.44
AmB-NE-F4	318.78 ± 2.38	0.234 ± 0.012	− 25.05 ± 1.56
AmB-NE-F5	296.65 ± 4.74	0.192 ± 0.011	− 24.90 ± 1.05

### A morphological study using TEM

**Fig. 3:**
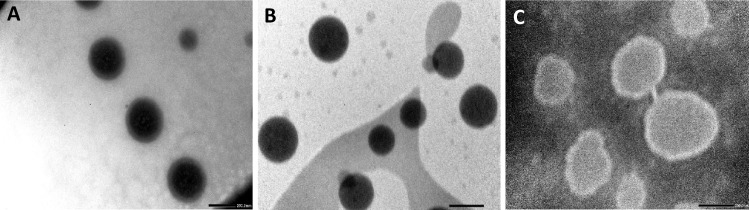
Transmission electron micrograph of **A** AmB-loaded-NE-F5, **B** blank NE, and **C** AmB-NE-DMN-F5. Scale bar, 200 nm

TEM is one of the most popular methods to characterize the morphology and droplet size of NEs. In this method, negative staining was used to enhance the contrast of the globular morphology. It is an established theoretical concept that maximum adherence of the nano-globules on the surface of the fungal cell increases the fungal killing [50]. A meaningful effort has been made to develop a small size of NE (< 320 nm), in order to treat cutaneous fungal infections when applied transdermally and intradermally via DMN. The approximate spherical nanoglobules of the optimized AmB-NE-F5, drug-free NE, and AmB-NE-DMN-F5 are given in Fig. 3A, B, and C, respectively. The illustrative microphotograph of the drug-loaded formulation revealed a dark black globule due to the dissolved form of the loaded drug. The TEM image of AmB-NE indicated monodispersed oil droplets with sizes < 300 nm, which further ascertained the results evaluated using the light scattering technique.

### Stability studies

The selected nanoemulsion (AmB-NE-F5) that showed good physicochemical properties was chosen to stability study for 15 days at 4 °C ± 2 °C (under refrigeration) and 25 °C ± 2 °C (room temperature). The droplet size, PDI, and zeta potential were found to be maintained without significant changes (see Table 4). The visual appearance of NE was homogenous, monophasic, and viscous with a yellowish color. In the current study, the presence of PVA and PVP polymers in the nanoemulsion played a significant role in the stability and thus retarding coalescence of individual droplets.

**Table 4:** Stability study data of optimized formulation (AmB-NE-F5)

Storage conditions	Days	Droplet size (nm)	PDI	Zeta potential (mV)
4 °C ± 2 °C (under refrigeration)	0	312.94 ± 4.47	0.220 ± 0.027	− 15.38 ± 1.05
	7	320.88 ± 4.80	0.250 ± 0.014	− 16.06 ± 0.089
	15	345.20 ± 2.82	0.223 ± 0.005	− 19.41 ± 0.027
25 °C ± 2 °C (room temperature)	0	312.94 ± 4.47	0.220 ± 0.027	− 15.38 ± 1.05
	7	316.62 ± 4.62	0217 ± 0.001	− 22.05 ± 1.08
	15	334.77 ± 7.88	0.259 ± 0.011	− 20.55 ± 0.26

### Fabrication and characterization of AmB-NE-loaded DMN

**Fig. 4:**
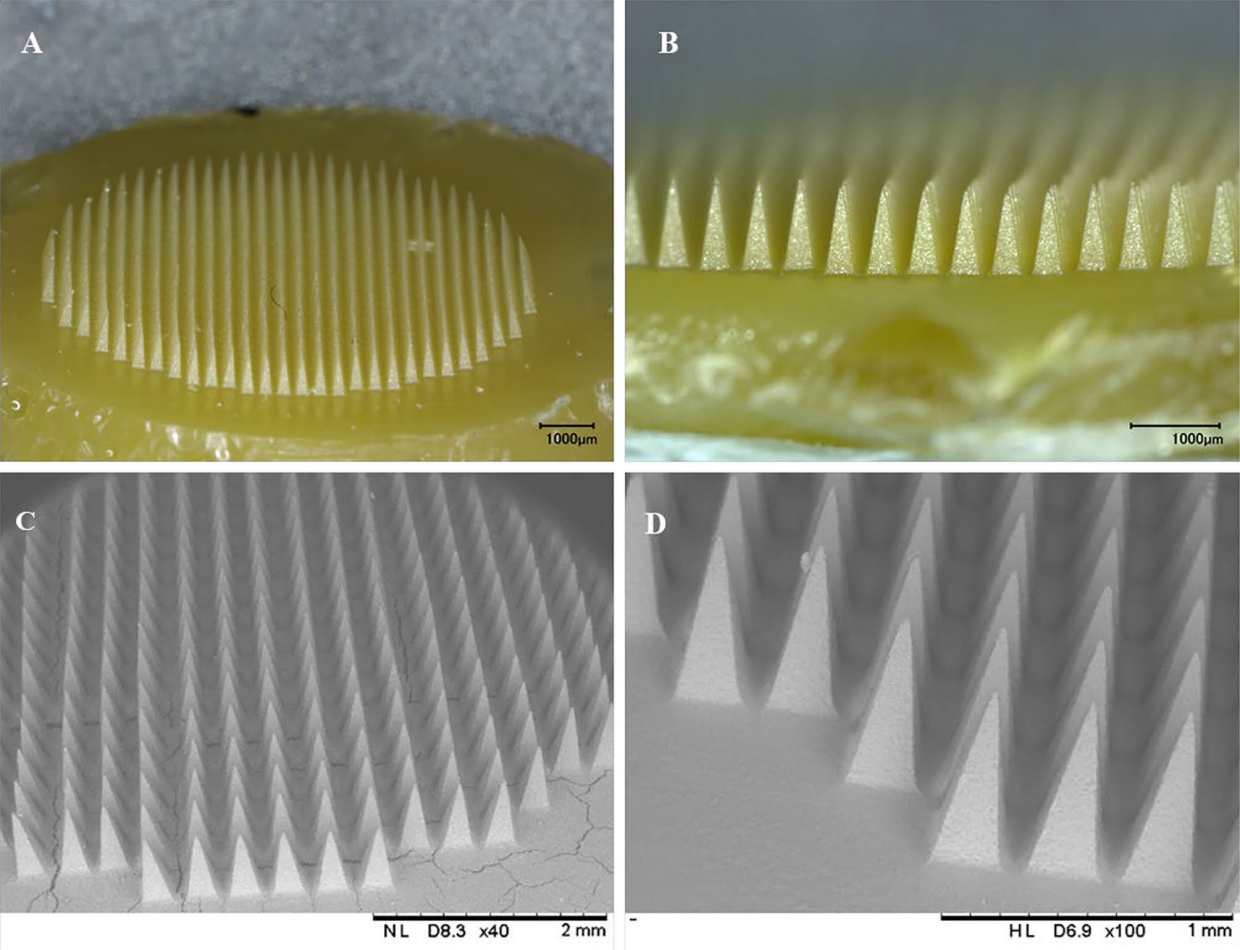
Digital microscopic images of AmB-loaded DMN arrays. **A** Complete AmB-NE-DMN. **B** AmB-NE-DMN arrays with high magnification. **C** Scanning electron micrograph of complete AmB-NE-DMN. **D** magnified image of AmB-NE-DMN arrays

The fabrication of AmB-NE-DMN arrays has been represented in Fig. 2. AmB-NE-DMN were prepared in a single-step centrifugation process of microneedle casting (MNs and baseplate were prepared using the same AmB-NE). The digital microscopic images (Fig. 4A and B) clearly indicated the formation of AmB-NE-DMN (600 arrays). Scanning electron microscopy was also used to further characterize the DMN (Fig. 4C and D). SEM images showed that NE-loaded DMN arrays were formed well structurally. The resulting needles measured 700 μm in height, displayed sharp tips. The amount of drug-loaded per patch, including baseplate, was 427 µg ± 0.11 μg (*n* = 3). The amount of drug in the tips of DMN patch was also determined as 35.0 ± 31.59 µg. Globule size is a significantly important stability parameter for NE during the finish formulation. Therefore, the selected DMN formulations (AmB-NE-DMN-F5) were evaluated for their droplet size, PDI, and zeta potential. The droplet size was observed as 374.45 ± 2.57 nm, PDI as 0.282 ± 0.015, and zeta potential as − 19.56 ± 2.66 mV. It indicates that AmB-loaded NE was stable after casting into DMN.

### Mechanical strength and Parafilm M^®^ insertion studies of AmB-NE-DMN

**Fig. 5:**
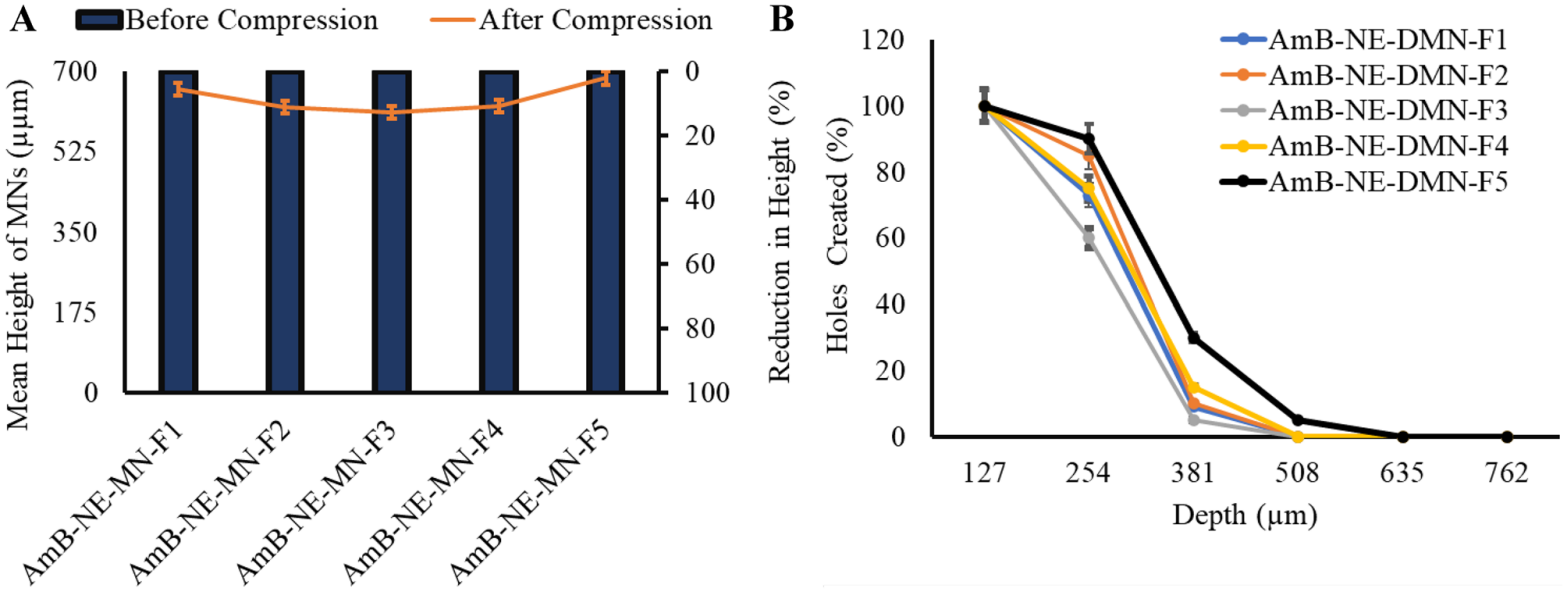
**A** Percentage reduction in the height of DMN upon exertion of the force of 32 N for 30 s (means ± SD, *n* = 3). **B** The percentage of holes created in Parafilm M^®^ layer and the corresponding approximate insertion depth, using an insertion force of 32 N (means ± SD, *n* = 3)

In order to assess whether DMN arrays are strengthened and do not break during skin penetration, the mechanical properties are usually evaluated. Mechanical properties of DMNs prepared using the different concentrations of PVP/PVA were assessed as described previously by Vora et al. [9]. Force was applied on the AmB-NE-DMN arrays to compress against a metal block. As a result, the height of MN was slightly compressed but, none of the MN fractured. All DMN formulations (see Table 2) prepared using different concentrations of PVA and PVP showed less than 10% reduction in the height of arrays after application of 32 N force [51]. Figure 5A reveals the mean percentage reduction in length of AmB-NE-DMN-F1 to AmB-NE-DMN-F5, which were found as 2%, 9%, 7.14%, 7.01%, and 1.4%, respectively. To check the insertion of the MN arrays, Parafilm M^®^ (an artificial membrane to mimic the skin) was used following the method described by Larrañeta et al. [52]. A light microscope was used to assess the number of holes created in each layer of Parafilm M^®^ sheet after applying AmB-NE-DMN array at 32 N force by Texture Analyser. Figure 5B shows the percentage of holes created in each Parafilm M^®^ layer after applying AmB-NE-DMN arrays. All AmB-NE-DMN arrays prepared from different PVA/PVP concentrations showed almost similar insertion profiles without any significant differences, whereas AmB-NE-DMN-F5 found excellent strength (1.4% reduction in length) and maximum penetration up to 4th parafilm layer (Fig. 5B). Considering the thickness of each layer of the Parafilm M^®^ membrane (127 μm), the insertion depth of AmB-NE-DMN-F5 was measured approximately 508 μm, which equates to > 73% of the needle height inserted (mean height 695 μm), without any reduction in height. Based on comparatively good mechanical and insertion profile of AmB-NE-DMN-F5 (prepared using 40% PVA and 60% PVP), it was chosen for further studies.

### Excised neonatal porcine skin insertion studies by optical coherence tomography

**Fig. 6:**
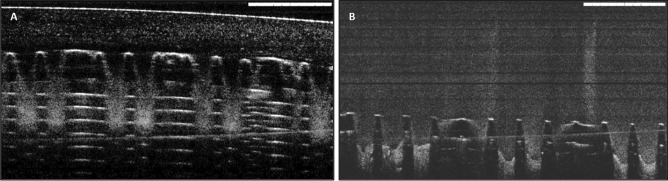
Optical coherence tomography images of **A** AmB-NE-DMN insertion in 8-layers of Parafilm M^®^ and **B** AmB-NE-DMN insertion in full-thickness neonatal porcine skin (scale bar: 1 mm)

OCT is a noninvasive optical imaging technique used to capture real-time images of the insertion of the AmB-NE-DMN arrays in the neonatal porcine skin and Parafilm M^®^ layers. The OCT images were examined using the imaging software ImageJ^®^ (National Institute of Health, Bethesda, USA). The scale of image files obtained was 1.0 pixel = 4.2 μm, thus allowing accurate measurements of the depth of DMN penetration, the distance between the MN baseplate and the *stratum corneum* [45]. AmB-NE-DMN patches revealed good insertion capability into neonatal porcine skin, reaching insertion depths of approximately 400–460 μm (Fig. 6A and B). While, in Parafilm M^®^, it was penetrated down up to the 4th layer (approximately 500 μm), as shown in Fig. 6C and D. Similar kinds of insertion results were reported previously in the insertion studies of polymeric MN into Parafilm M^®^ [53, 54]. These results indicated that AmB-NE-DMN was mechanically strong enough to penetrate the porcine skin.

### Dissolution of AmB-NE-DMN after insertion into porcine skin

**Fig. 7:**
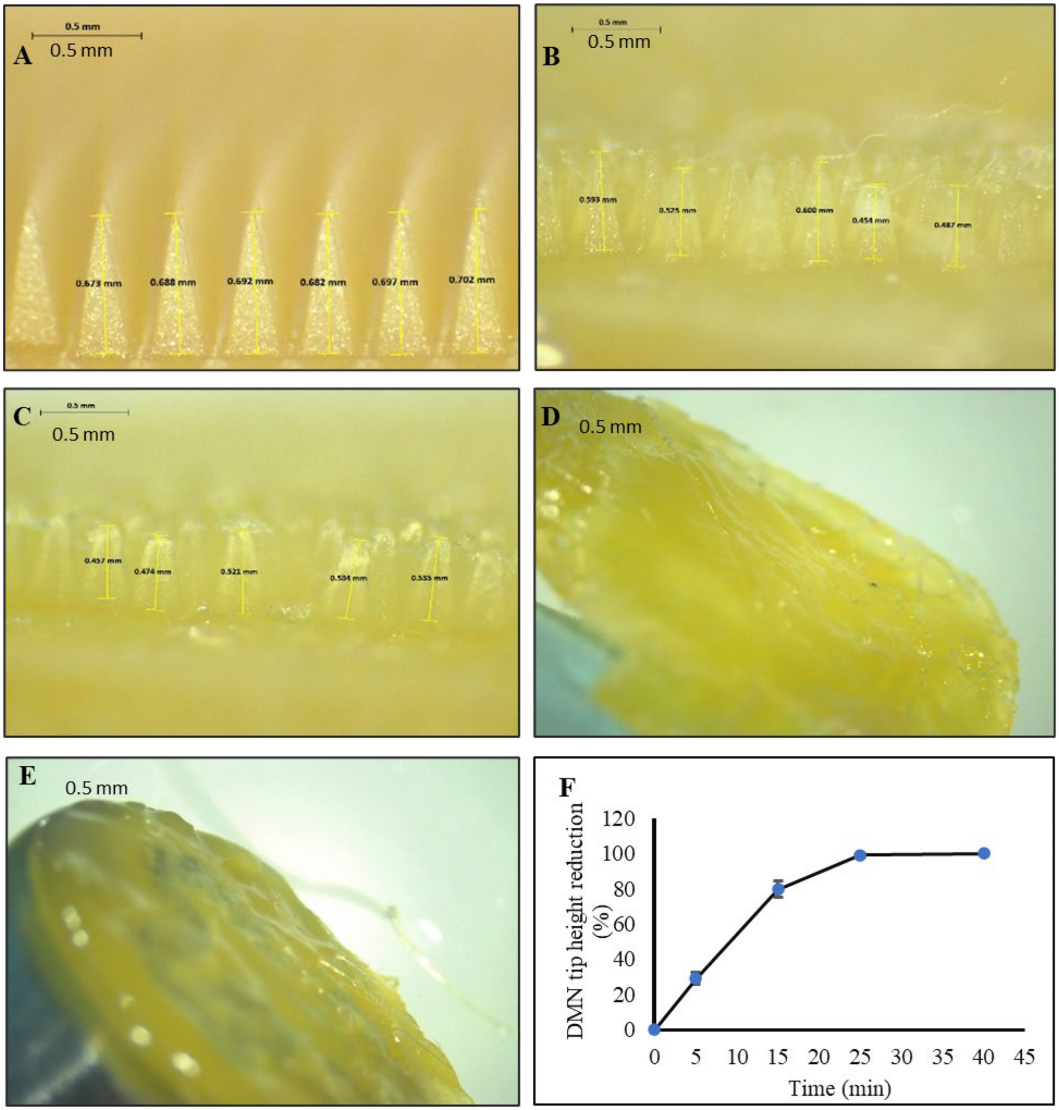
Representative digital micrographs of the dissolution of AmB-NE-DMN-F5 at specific time points **A** *T* = 0 min; **B** T = 5 min; **C** T = 15 min; **D** T = 25 min; **E** T = 40 min, following insertion into, and removal from excised neonatal porcine skin (scale bar: 0.5 mm). **F** The percentage of height reduction of DMN tips of finalized AmB-NE-MN-F5 after insertion into and removal from an excised full-thickness neonatal porcine skin at predetermined time intervals (*n* = 3)

This study was conducted with a view to anticipating the time required for MN array dissolution after inserting into full-thickness porcine skin. The AmB-NE-DMN were inspected before and after application by light microscopy (Fig. 7A–E) to determine the percentage height reduction of DMN tips (due to dissolution in the skin) versus time (Fig. 7F). The AmB-NE DMN F5 developed by using PVA (40% w/w) and PVP (60% w/w) illustrated 100% dissolution of needles within 25 min, as displayed in Fig. 7D. Similarly, the baseplate containing the AmB-NE was completely dissolved after 40 min, as indicated in Fig. 7E. These outcomes showed that this MN is rapid-dissolving for a shorter application time to deliver AmB-NE into the skin.

### Ex vivo porcine skin permeation of AmB from AmB-NE-DMN

**Fig. 8:**
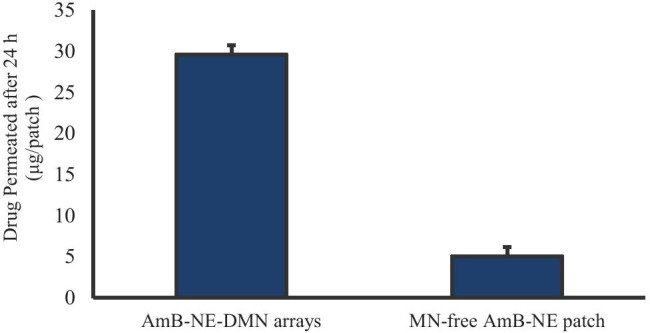
In vitro drug release profile of AMB-NE-DMN arrays versus MN-free AMB-NE patch (means + SD, *n* = 5)

The ex vivo neonatal porcine skin was investigated to study the permeation of AmB across the skin using Franz diffusion cells as reported previously [41] and trimmed to a thickness of 350 μm. Then, AmB-NE-DMN-F5 was inserted into the skin using manual pressure for 30 s, and the release of the drug was monitored at different time intervals (up to 24 h). For comparison, MN-free AmB-NE patches were also applied over the skin. Following application of the DMN and MN-free patches for 24 h, a large quantity of AmB was diffused from AmB-NE-DMN arrays as compared to MN-free AmB-NE patches, as shown in Fig. 8. It was indicated that AmB-NE-DMN arrays displayed higher ex vivo skin permeation compared with MN-free AmB-NE patches over 24 h. The DMN containing nanosized droplet probably increases the delivery of the drug by permeating the *stratum corneum* barrier and through MN induced micro-conduits into the skin, thereby allowing systemic drug absorption.

It is well reported that the delivered dose from MN is generally restrained between micro to lower milligram range, and it depends upon percentage loading of the drug and the total array volume (i.e., shape, size, and density of MNs) [7, 55].

### Ex vivo deposition studies of AmB-NE-DMN into excised porcine skin

Full-thickness neonatal porcine skin obtained from stillborn piglets was used. The main objective of this study was to explore the possibility of delivering the model drug using NE-DMN. The AmB NE loaded DMN arrays were manufactured with drugs in the needle tips (to deliver intradermally) and in the baseplate (to deliver transdermally). In this way, the drug can be delivered from the needle tips and consequently from the baseplate. The drug confined in the baseplate can permeate through the pores created by needle tips [56]. Here, AmB-NE-DMN patches were inserted into porcine skin. After 24 h application, the residual patch was completely removed, and the application site was thoroughly rinsed with PBS. The skin tissue sample was cut by a Scalpel from that portion of the skin where the AmB-NE-DMN was applied. The tissue samples were bead-milled using TissueLyser with DMSO, sonicated as per described method, and the supernatants were analyzed using the validated HPLC method. Following the application of the DMN for 24 h, 111 ± 48.4 μg**/**patch AmB was deposited from AmB-NE-MN arrays into the skin which is 26% ± 11% delivery efficiency from total patch dose. Therefore, the results manifest that the AmB-NE-DMN arrays undoubtedly help the delivery of the AmB through the skin to obtain improved intradermal delivery.

Fungal skin diseases are very complex in nature. A desirable drug delivery system should be able to improve the accumulation of the active drugs in the target tissue. Drug deposition, as well as penetration across the skin, differs on a great range of factors. One of them is the pathway that drugs take to penetrate the dermis. The biggest barrier to penetration of the skin is the *stratum corneum*. This skin construct has a “brick and mortar” structure of corneocytes (the “bricks,” mainly composed of hydrated keratin) and, fatty acids, multilamellar layers of ceramides, cholesterol and cholesterol esters (the “mortar”) [57]. The epidermis is formed into clusters or columns of cells, split up from each other by furrows (“canyons”) loaded with lipids that traverse the entire epidermis, extending the basal layer near the dermis. There is the possible role of NE-like lipidic system penetrate deeper through the canyons than through the cell clusters [58]. DMN first assists the NE to deposit into the dermis/epidermis region by physically breaking the *stratum corneum* barrier. The incorporation of NE avoids the crystallization of insoluble drugs by dissolving them in a lipid matrix. NE vesicles are ultra-flexible and their lipidic components are capable of penetrating the epidermis/dermis interface that could allow deeper and better cutaneous drug distribution for effective anti-infective properties [58]. Therefore, these NE and DMN based synergistic approaches will pave the way for effective drug penetration and deposition into the skin.

### In vitro antifungal activities

**Fig. 9:**
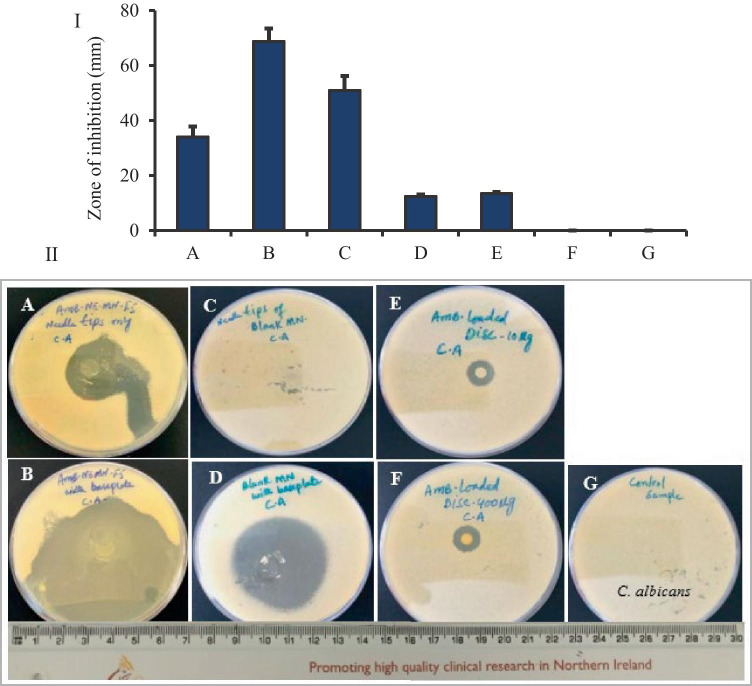
(I) Graph of the zone of inhibition values (mm) versus groups. The data represents the mean + SD (*n* = 4). (II) Photographs of the zone of inhibition against *C. albicans*: (**A**) tips of AmB-NE-DMN arrays, (**B**) AmB-NE-DMN arrays with baseplate, (**C**) tips of blank-NE-DMN arrays, (**D**) blank-NE-DMN arrays with baseplate, (**E**) AmB-loaded disk (10 µg), (**F**) AmB-loaded disk (400 µg), (**G**) control without any treatment

Antifungal activity of AmB-NE-DMN-F5 (optimized formulation) and AmB-loaded discs were assessed against *Candida albicans* (*CA*). The *CA* infections are most common superficial cutaneous fungal infections. It is also the cause of sepsis, wound infections, and pneumonia, particularly in immunosuppressed patients. This *CA* infection can invade deep tissues as well as the systemic blood circulation, which advances to life-threatening systemic infection, particularly in those immunocompromised patients [13]. To test antifungal activities of these developed NE-DMN formulations with AmB, the study was divided into seven groups such as A, B, C, D, E, F, and G (A = tips of AmB-NE-MN-F5 arrays (approx. 35 µg of AmB), B = AmB-NE-DMN-F5 arrays with a baseplate (427 µg), C = tips of blank-NE-DMN arrays, D = blank, and G = Control without any treatment) [59]. Figure 9 (I) shows the zone of inhibition (ZOI) of the in vitro antifungal activity test after 72 h and the data represent the mean ± SD, (*n* = 4). Figure 9 (II) shows the photographic images of ZOI against *Candida albicans.* ZOI values for group B (68.75 ± 4.79 mm) were significantly greater than group A (34.0 ± 3.92 mm), while ZOI values for group D (51.0 ± 5.29 mm). The inhibition zone of growth was not a regular circle, probably because the dissolution of AmB as well as faster distribution of oily NE from the dissolving polymeric matrices and aqueous agar plates promoted the release of AmB into a larger area. There is no inhibition of CA growth in blank DMN from group C, which is similar with untreated group (G), indicating that polmyers (PVA and PVP) could not exert antifungal effects. However, ZOI values for group-E (12.5 ± 0.58 mm) was not sufficiently different from group-F (13.5 ± 0.58 mm), indicating that AmB-NE-DMN-F5 formulation has excellent activity against *C. albicans* as compared to pure AmB-loaded disk. This sufficiently high activity of DMN may be due to the synergistic activity of oil (Campul-MCM C-8) used in the formulation development of NE. Group D (blank-NE-DMN arrays with baseplate) also showed ZOI, which confirmed the inherent antifungal activity of oil as reported previously [23]. Group G was control sample without any MN and disc treatments to ascertain the growth of *CA.* This study demonstrated the synergistic fungal killing ability of AmB with NE from DMN.

## Conclusion

The NE of the highly hydrophobic drug was successfully optimized and incorporated into DMN arrays to penetrate the skin and dissolve rapidly in the skin to achieve adequate drug permeation and deposition. NE loaded DMN arrays were formulated with the model drug, AmB. The stability of the drug-loaded NE was confirmed by using a particle size analyzer. These self-dissolving MN fabricated from PVA/PVP showed good mechanical strength for enhanced intra- and trans-dermal drug delivery. Novel NE-loaded DMN system may be able to deliver a wide range of lipophilic compounds intradermally, especially those available in liquid form. As per obtained results, AmB NE-loaded DMN could provide the synergistic antifungal effect to ensure its efficacy against *Candida albicans*. In conclusions, this proof-of-concept work, therefore, represents meaningful advancement in the usage of DMN technologies in combination with nanoemulsion for delivery of lipophilic drugs into the viable skin (epidermis and dermis) layers for maximum therapeutic achievement and better patient compliance. Further preclinical studies are needed to translate this concept for clinical application.

## Data Availability

All data generated or analyzed during this study are included in the article.
